# Supplementary figures and images for: TgIF2K-B Is an eIF2α Kinase in Toxoplasma gondii That Responds to Oxidative Stress and Optimizes Pathogenicity
Source: mBio. 2021 Jan 26;12(1):e03160-20. doi: 10.1128/mBio.03160-20 (PMC7858062; doi:10.1128/mBio.03160-20)

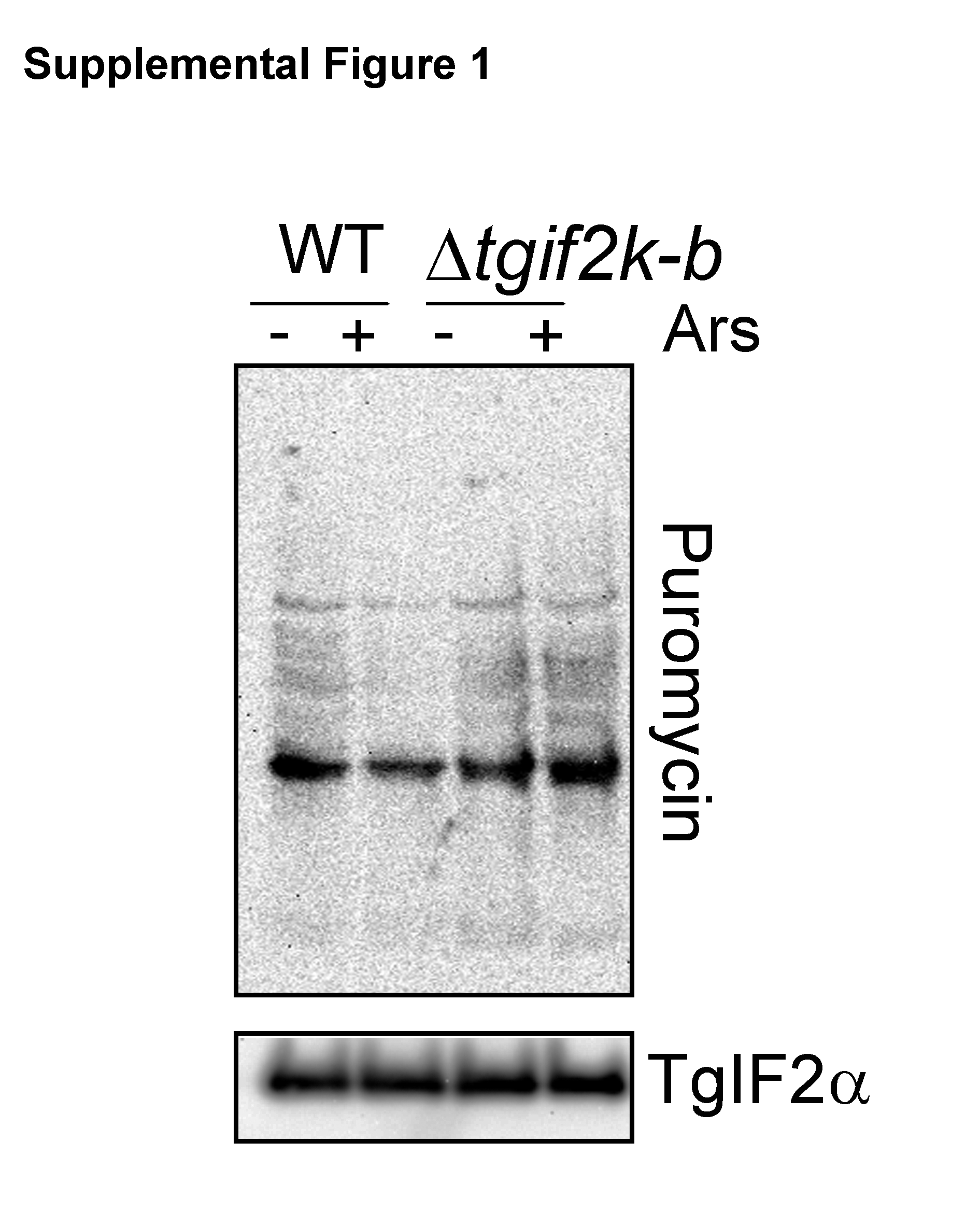

Supplement: FIG S1 [file mBio.03160-20-sf001.tif]

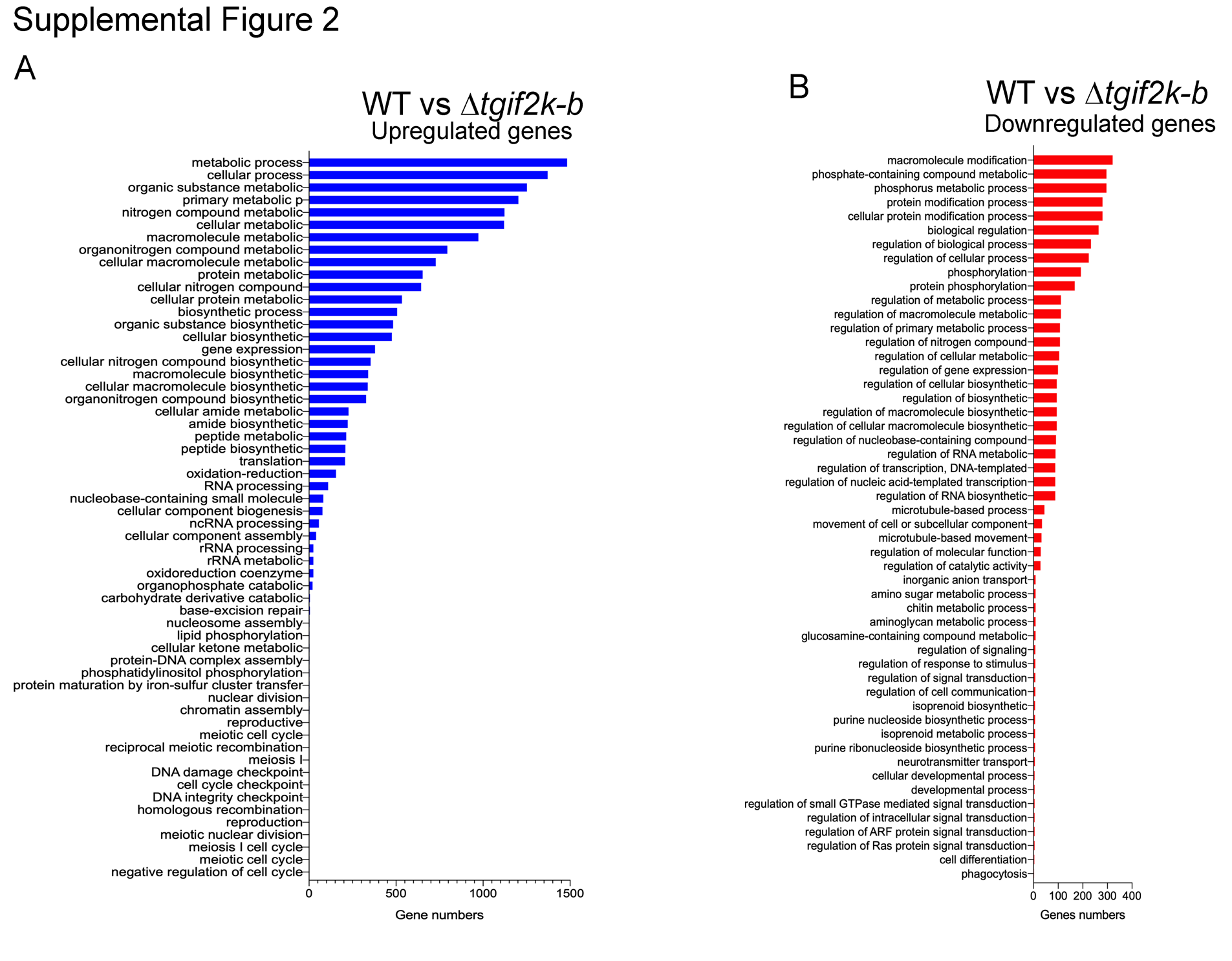

Supplement: FIG S2 [file mBio.03160-20-sf002.tif]

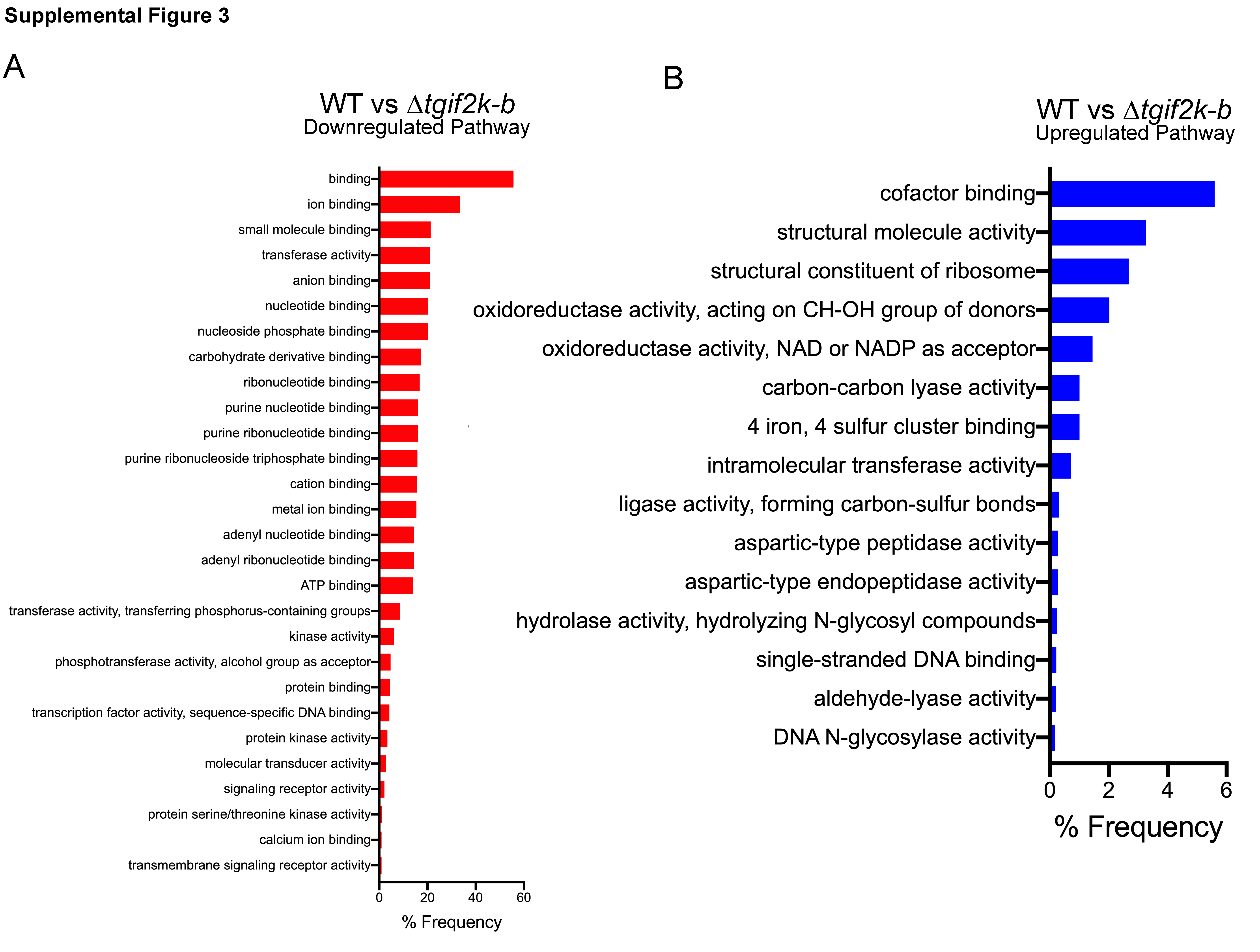

Supplement: FIG S3 [file mBio.03160-20-sf003.tif]

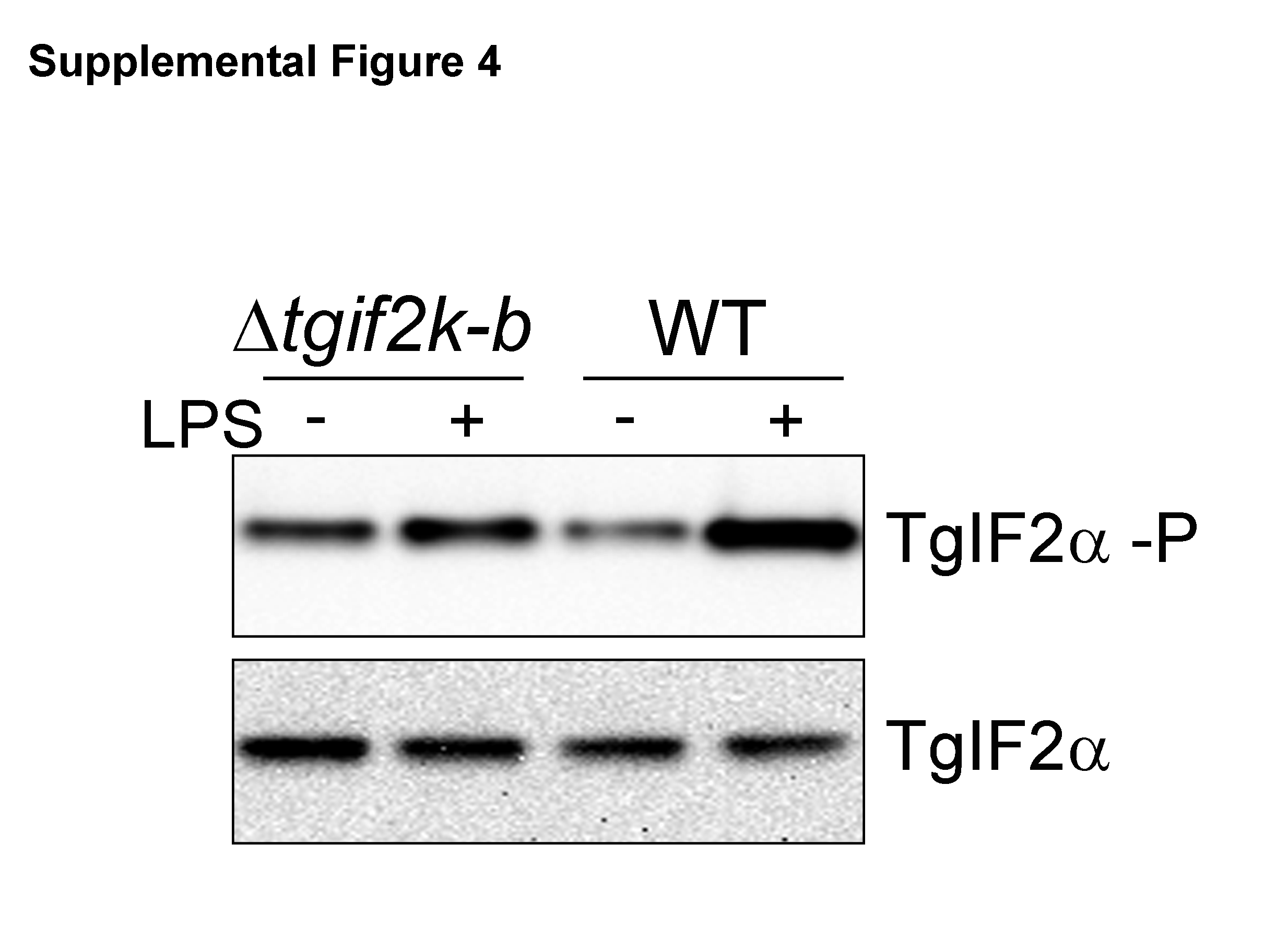

Supplement: FIG S4 [file mBio.03160-20-sf004.tif]
